# Frequency of resistance to first-line antiretroviral therapy observed among HIV patients

**DOI:** 10.12669/pjms.38.7.6144

**Published:** 2022

**Authors:** Feroz Khan, Muhammad Bilal, Muhammad Yaseen Khan, Mian Fareezuddin

**Affiliations:** 1 Feroz Khan, MBBS. Lady Reading Hospital, MTI, Peshawar, Pakistan; 2 Muhammad Bilal, FCPS, MRCPI. Lady Reading Hospital, MTI, Peshawar, Pakistan; 3 Muhammad Yaseen Khan, FCPS, FRCP. Lady Reading Hospital, MTI, Peshawar, Pakistan; 4 Mian Fareezuddin, MCPS. Federal Public Service Commission, Pakistan

**Keywords:** HIV Infection, Antiretroviral Failure, Resistance, Risk Factors

## Abstract

**Objectives:**

This study aimed to assess the frequency of first-line antiretroviral therapy (ART) resistance among HIV patients and to identify the factors affecting the drug resistance.

**Methods:**

A cross-sectional study was conducted over a sample of 162 HIV-positive patients attending the Medicine Department of Lady Reading Hospital, Peshawar-Pakistan, from July 2020 to January 2021. Blood samples were collected for phylogenic profiles to determine first-line antiretroviral therapy resistance.

**Results:**

The frequency of ART resistance was detected in 64.8% of the enrolled HIV patients. Factors such as patient age, gender, comorbidities, and smoking status had no significant impact on drug resistance. While only body mass index (BMI) significantly affected ART resistance among HIV patients. The drug resistance mutations M184V and K103N were detected in the nucleoside reverse transcriptase inhibitors (NRTIs) and non-nucleoside reverse transcriptase inhibitors (NNRTIs), respectively, whereas the mutations G73SC and I47VA were detected in the protease inhibitors (PIs).

**Conclusion:**

There is a high frequency of resistance to first-line antiretroviral therapy among HIV patients presenting to the selected healthcare facility in Peshawar. Furthermore, we found no significant factors impacting ART resistance among HIV patients other than BMI.

## INTRODUCTION

Acquired immunodeficiency syndrome (AIDS)-associated problems resulting from the HIV contributes to a significant number of mortalities every year. According to UNAID HIV and AIDS statistics (2020), globally, 37.7 million people have been infected and living with HIV^1^, of which approximately 1.5 million were newly infected, and over 680,000 of them died from AIDS-associated ailments.^1^ The estimated prevalence of HIV in Pakistan is 0.1%, i.e., 183,705 people are living with HIV.^2^

In developing countries, specifically Pakistan, the socioeconomic burden, including high poverty, illiteracy, unemployment, and increased healthcare cost, are likely to enhance the transmission of HIV. Although HIV is widespread in every population subgroup, it is spreading more rapidly among the male escorts as compared to female, demonstrating variety in local sexual practices.^3^ Furthermore, HIV is on the rise among intravenous drug users (IDUs), i.e., 38.4%.^4^ In many parts of Pakistan, IDUs involved in sexual activities with MSWs and transgender escalate HIV transmission, consequently following the Asian Epidemic Model.^5^,^6^

As per the HIV/AIDS prevention policies, early diagnosis and subsequent initiation of antiretroviral therapy (ART) remarkably reduces morbidity and mortality among patients infected with HIV. The timely provision of ART ensures appropriate management of opportunistic infections, preventing secondary transmission and associated complications^6^, leading to improved health and prolonging the life expectancy among the people living with HIV.^7^,^8^ Though it is highly recommended to initiate the therapy before the infection begins, the delayed or interrupted HIV treatment initiations has been widely observed in developed and developing countries.^9^ The treatment onset and compliance vary with respect to culture and traditions worldwide.^7^

As of June 2020, 28.2 million people are receiving ART drugs, i.e., 73% of the total people living with HIV.^1^ While in Pakistan, only 10% of HIV patients are receiving ART with extremely low treatment compliance.^4^ However, with the increasing accessibility of HIV treatment, the rate of treatment discontinuation has also increased, resulting in a subsequent decline in the CD4+ T cell counts.^10^ Such patients are at higher risk of AIDS, morality and are a probable source of HIV transmission.^11^ Furthermore, ART efficacy tends to compromise with the development of HIV drug resistance (HIVDR).^12^ The low fidelity of HIV reverse transcriptase, rapid viral replication, and the selective pressure of antiretroviral drugs promote HIVDR.^13^

There has been a scarcity of data from Pakistan addressing the issue. Therefore, the current study aimed to assess the frequency of resistance to first-line antiretroviral therapy among HIV patients and its association with demographic characteristics.

## METHODS

This cross-sectional study was conducted at the Department of Medicine, Lady Reading Hospital, Peshawar-Pakistan, from July 2020 to January 2021. A sample size of 162 was calculated using the WHO sample size calculator with a 6.5% margin of error, 95% confidence level, the prevalence of first-line antiretroviral resistance in HIV patients of 23.1%.^14^ Prior to inclusion, written informed consent was obtained from each patient or next of kin after explaining the study purpose. The study protocol was approved by the ethical review committee of Lady Reading Hospital (Reference no; 21/LRH Dated: 04/12/2018), and patient confidentiality was maintained.

All confirmed seropositive HIV patients between 18 to 70 years of age, both men and women receiving 1^st^ line anti-retroviral therapy (ART), were included in the study, while non-consenting patients and HIV seropositive patients on 2^nd^ line antiretroviral therapy were excluded.. The patient’s detailed history, including age, gender, BMI, comorbid conditions, smoking status, viral load, and first-line antiretroviral response, were recorded using a structured questionnaire. Blood samples were drawn for phylogenetic profiling. The resistance to first-line antiretroviral therapy was determined on the basis of viral load.

Data were analyzed using SPSS version 22.0, frequency and percentages were used to present categorical variables like gender, age groups, hypertension, diabetes mellitus, smoking status, and ART resistance. Mean and standard deviation was used for continuous variables like age and BMI. Post-stratification Chi-square test to see the effect modification by age, gender, body mass index, smoking, diabetes mellitus, and hypertension, where a p-value < 0.05 was considered statistically significant.

### Ethical Approval

(Ref: 21ILRH, Dated 04-12-2018).

## RESULTS

Out of the total, 52.5% of HIV patients were females, and 47.5% were males. The mean age of these patients was 45.44 ± 5.0 years. The frequency of ART resistance was observed among 64.8% of the total HIV patients (Table-I). There was no significant effect of age, gender, smoking status, presence of diabetes, or hypertension on the HIV drug resistance. While only BMI was significantly associated with resistance status (p=0.023) (Table-II). Of the genotyped sample, the drug resistance mutations M184V and K103N were detected in the NRTIs, and NNRTIs, respectively, whereas the mutations G73SC and I47VA were detected in the PIs.

**Table-I:** Baseline characteristics of the patients.

Variables		N(%)
Age; mean ± SD (Years)		45.44 ± 5.0
Gender	Male	77(47.5)
	Female	85(52.5)
Diabetes Mellitus	Yes	64(39.5)
	No	98(60.5)
BMI (kg/m^2^)	Normal	57(35.2)
	Overweight	51(31.5)
	Class-I Obesity	38(23.5)
	Class-II Obesity	16(9.9)
Hypertension	Yes	88(54.3)
	No	74(45.7)
Smoking	Yes	80(49.4)
	No	82(50.6)
Resistant	Yes	57(35.2)
	No	105(64.8)

**Table-II:** Effect of baseline characteristics on HIV drug resistance.

Variables		Resistant		p-value

		Yes	No	
Gender	Male	32(56.1)	45(42.9)	0.106
	Female	25(43.9)	60(57.1)	
Diabetes Mellitus	Yes	22(38.6)	42(40.0)	0.861
	No	35(61.4)	63(60.0)	
BMI	Normal	15(26.3)	42(40.0)	0.023*
	Overweight	17(29.8)	34(32.4)	
	Class-I Obesity	21(36.8)	17(16.2)	
	Class-II Obesity	4(7.0)	12(11.4)	
Hypertension	Yes	34(59.6)	54(51.4)	0.316
	No	23(40.4)	51(48.6)	
Smoking	Yes	30(52.6)	50(47.6)	0.542
	No	27(47.4)	55(52.4)	

* p<0.05 is considered statistically significant.

## DISCUSSION

In the present study, we evaluated drug resistance and the phylogenetic profile of HIV patients presenting at the Lady Reading Hospital, Peshawar. To the best of our knowledge, this has been the first attempt from Peshawar, to address drug resistance among HIV patients referred through clinicians with suspected clinical or immunological failure.

The frequency of drug-resistant variants was 35.2% in the studied population, which is comparatively high than a study conducted in China reporting 19.5% patients with drug resistance variants.^15^ Similarly, an Italian study reported that around 21.8% of HIV patients were resistant to one drug class, 24.6% to two, and 12.9% to three drug classes.^16^ The high drug resistance reported in the present study could either be related to non-adherence to ART or late recognition of treatment failure.

The M184V mutation was more frequently observed than other mutations, determined as the major reason drug resistance to NRTIs. These findings were similar to other parallel studies.^17^,^18^ Furthermore, for NNRTIs, K103N was the most common mutated sequence, which is comparable to other studies^15^,^19^, it is known to compromise efficacy of Nevirapine and Efavirenz. Additionally, the low genetic barrier of the NNRTI drugs permit resistance to these drugs.^20^ For protease inhibitors (PIs), G73SC and I47VA were common mutations observed. Unlike the other classes of ART drugs, the displayed resistance to PIs in the present study was contrast to other similar study.^21^ G73SC mutation considerably reduces the nelfinavir and saquinavir susceptibility, while high-level resistance to lopinavir and fosamprenavir and low/intermediate resistance to the remaining PIs except for atazanavir and saquinavir, has been contributed by I47VA mutation.^22^

We found no significant factors influencing HIV drug resistance except BMI (p=0.023). Whereas Sebastião et al. studied the factors influencing HIV drug resistance among pregnant females, they found a high frequency of drug resistance among the pregnant HIV females with other comorbidities, living in rural areas, and those with high educational status.^23^ Similarly, other studies also confirm that patients’ age, residence, education, occupational status, comorbid conditions, and HIV-1 subtypes are the significant factors related to ART failure.^24^,^25^

### Limitations of the Study

Among the major limitations of the present study was its cross-sectional design and devoid generalizability; these outcomes cannot be generalized for the entire Pakistani population, as the study included only the HIV-positive patients from Peshawar attending the study site for treatment. The patient’s residence and educational status weren’t considered, which might have been significant influencers of drug resistance. Furthermore, the resistance testing was not performed prior to initiation of ART, and treatment adherence was not established; hence it is not imperative to say that all the resistance mutations occurred after ART initiation or because of non-adherence to ART.

## CONCLUSION

There is a high frequency of ART-resistant variants among enrolled HIV patients, which highlights the importance of timely recognition of treatment failure. The mutations M184V and K103N were detected in the NRTIs and NNRTIs, respectively, whereas the mutations G73SC and I47VA were detected in the PIs. Furthermore, the provision and accessibility of genotypic testing remain a challenge; less expensive approaches for the assessment of viral load are highly essential in a country like Pakistan.

### Authors’ Contribution:

**FK:** Is responsible for the study design, data collection and write up.

**MB:** Contributed to the literature search and manuscript editing.

**MYK:** Is responsible for data analysis and literature search of the manuscript.

**MF:** Contributed to the write up and editing of the study.

All the authors are equally responsible for the integrity of the research work.
